# Are anti-ganglioside antibodies associated with proventricular dilatation disease in birds?

**DOI:** 10.7717/peerj.3144

**Published:** 2017-04-11

**Authors:** Jeann Leal de Araujo, Ian Tizard, Jianhua Guo, J Jill Heatley, Aline Rodrigues Hoffmann, Raquel R. Rech

**Affiliations:** 1 Department of Veterinary Pathobiology, Texas A&M University, College Station, TX, United States; 2 Department of Small Animal Clinical Sciences, Texas A&M University, College Station, TX, United States

**Keywords:** PDD, Psittaciformes, Pathogenesis

## Abstract

The identification of Parrot bornaviruses (PaBV) in psittacine birds with proventricular dilatation disease (PDD) has not been sufficient to explain the pathogenesis of this fatal disease, since not all infected birds develop clinical signs. Although the most accepted theory indicates that PaBV directly triggers an inflammatory response in this disease, another hypothesis suggests the disease is triggered by autoantibodies targeting neuronal gangliosides, and PDD might therefore resemble Guillain-Barré Syndrome (GBS) in its pathogenesis. Experimental inoculation of pure gangliosides and brain-derived ganglioside extracts were used in two different immunization studies. The first study was performed on 17 healthy chickens (*Gallus gallus domesticus*): 11 chickens were inoculated with a brain ganglioside extract in Freund’s complete adjuvant (FCA) and six chickens inoculated with phosphate-buffered saline. A second study was performed five healthy quaker parrots (*Myiopsitta monachus*) that were divided into three groups: Two quaker parrots received purified gangliosides in FCA, two received a crude brain extract in FCA, and one control quaker parrot received FCA alone. One chicken developed difficult in walking. Histologically, only a mild perivascular and perineural lymphocytic infiltrate in the proventriculus. Two quaker parrots (one from each treatment group) had mild lymphoplasmacytic encephalitis and myelitis. However, none of the quaker parrots developed myenteric ganglioneuritis, suggesting that autoantibodies against gangliosides in birds are not associated with a condition resembling PDD.

## Introduction

Although parrot bornaviruses (PaBV) are consistently isolated from psittacine birds with proventricular dilatation disease (PDD) (Honkavuori et al., 2008; Kistler et al., 2008), the pathogenesis of this condition still remains unclear since not all infected birds develop clinical signs. Neurotropism is an important characteristic of PaBV and lymphoplasmacytic encephalomyelitis and ganglioneuritis, particularly prominent in the enteric nervous system, are the hallmark lesions of PDD (Rinder et al., 2009; Staeheli, Rinder & Kaspers, 2010; Weissenbock et al., 2009). Therefore, PaBV infection can cause neurologic and/or gastrointestinal signs, including lethargy, ataxia, regurgitation, emaciation and ultimately culminating in death (De Kloet, Kerski & de Kloet, 2011; Kistler et al., 2010; Payne et al., 2011). As the name suggests, the dilatation of the proventriculus is the most prominent macroscopic lesion of this disease (Gancz et al., 2009; Rinder et al., 2009).

Several mechanisms have been proposed to explain how PaBV causes ganglioneuritis as well as the encephalomyelitis in birds (Piepenbring et al., 2012). The pathogenesis of PDD has been linked to two main hypotheses: the first and most accepted theory states that PaBV spreads throughout the nervous system and directly triggers the inflammatory and immunological changes that lead to damage in the central nervous system (CNS), the enteric nervous system and peripheral nerves (Piepenbring et al., 2016). The second hypothesis proposes the pathogenesis of PDD in psittacine birds is caused by the production of autoantibodies targeting components of the nervous system (Rossi, Crosta & Pesaro, 2008), a similar mechanism to an autoimmune condition of humans called Guillain–Barré syndrome (GBS).

GBS is the most common cause of acute flaccid paralysis in humans since the near eradication of poliomyelitis (Nachamkin, Allos & Ho, 1998). The disease results in a demyelinating neuropathy that affects the peripheral nervous system (PNS) and causes symmetrical limb weakness that may lead to paralysis in 2–3 weeks (Van den Berg et al., 2014). The pathogenesis of GBS and other autoimmune diseases such as, acute disseminated encephalomyelitis and neuromyelitis optica, is associated with molecular mimicry. In this case, bacteria (e.g., *Campylobacter jejuni*) or viruses (e.g., Cytomegalovirus, Zika virus, Chikungunya virus, Epstein-Barr virus) mimic the structure of a specific host antigen and so triggers an immune reaction against tissue containing the cross-reacting antigen, such as neuronal gangliosides (Nyati & Nyati, 2013; Dos Santos et al., 2016; Venkatesan & Benavides, 2015; Oehler et al., 2015). These complex glycosphingolipids are expressed in abundance on neurons of the CNS and PNS (Yu et al., 2008), and have an important role in cellular interactions. When targeted by auto-antibodies, neuronal damage occurs, leading to the development of GBS (Yu, Usuki & Ariga, 2006).

This study aimed to evaluate the relationship between the experimental inoculation of gangliosides or crude nervous tissues and the development of neurologic disease in chickens (*Gallus gallus domesticus*) and PaBV-free quaker parrots (*Myiopsitta monachus*) that could resemble PDD.

## Material and Methods

### Experimental design

Two experiments were performed. The first experiment aimed to analyze whether or not the inoculation of gangliosides in an avian species (chickens) could lead to the development of neurological or gastrointestinal signs that could resemble PDD. Only histologic examination was performed in this experiment. A second experiment was performed in order to determine if the inoculation of crude nervous tissue or purified gangliosides in a psittacine species (quaker parrots) could lead to neurological or gastrointestinal disease, resembling those seen in cases of PDD. In this experiment, blood samples were collected in order to evaluate anti-ganglioside antibody and fibrinogen levels. Samples were also collected for histologic examination.

### Inoculum preparation and purification

Gangliosides were extracted from brain, spinal cord, sciatic and brachial nerves, and nerve of Remak from ten healthy chickens, and three healthy PaBV-free quaker parrots, using a modified technique (Svennerholm & Fredman, 1980) adapted for this purpose by Pesaro (Pesaro, 2008).

### Thin layer chromatography (TLC)

The isolation of brain gangliosides was confirmed using a thin-layer chromatography (Scandroglio et al., 2009). Given the lack of commercially available avian gangliosides, a bovine ganglioside mixture (Millipore, Billerica, MA, USA) was used as the standard for the TLC analysis. The solvent system utilized for this purpose was composed of 50:42:11 volume of chloroform/methanol/0.2% aqueous CaCl_2_. After separation, gangliosides were stained using Ehrlich’s reagent, prepared with 6 g of p-dimethylaminobenzaldehyde in 50 ml of 37% hydrochloric acid and 50 ml of 95% ethanol.

### Experimental animals

The experiments described here were performed under animal use protocol number IACUC 2014-0006, approved by the Texas A&M University Institutional Animal Care and Use Committee. To determine if the inoculation of brain gangliosides into an avian species could induce neurologic disease, we performed a preliminary study using 17 healthy female, white, leghorn chickens, between 10 and 15 weeks old, and vaccinated for Marek’s disease originated from Texas A&M University Poultry Science Center, which were divided into two groups: the first group was comprised of 11 chickens (C1–C11), inoculated with 1 ml of purified gangliosides and FCA in the pectoral muscle. The second group consisted of six chickens (C12–C17) inoculated only with phosphate-buffered saline (PBS) and served as controls. All chickens were inoculated twice with 31 days between each inoculation, as performed before by Pesaro (2008). The chickens that did not present clinical signs were euthanized 62 days post inoculation (dpi).

**Figure 1:**
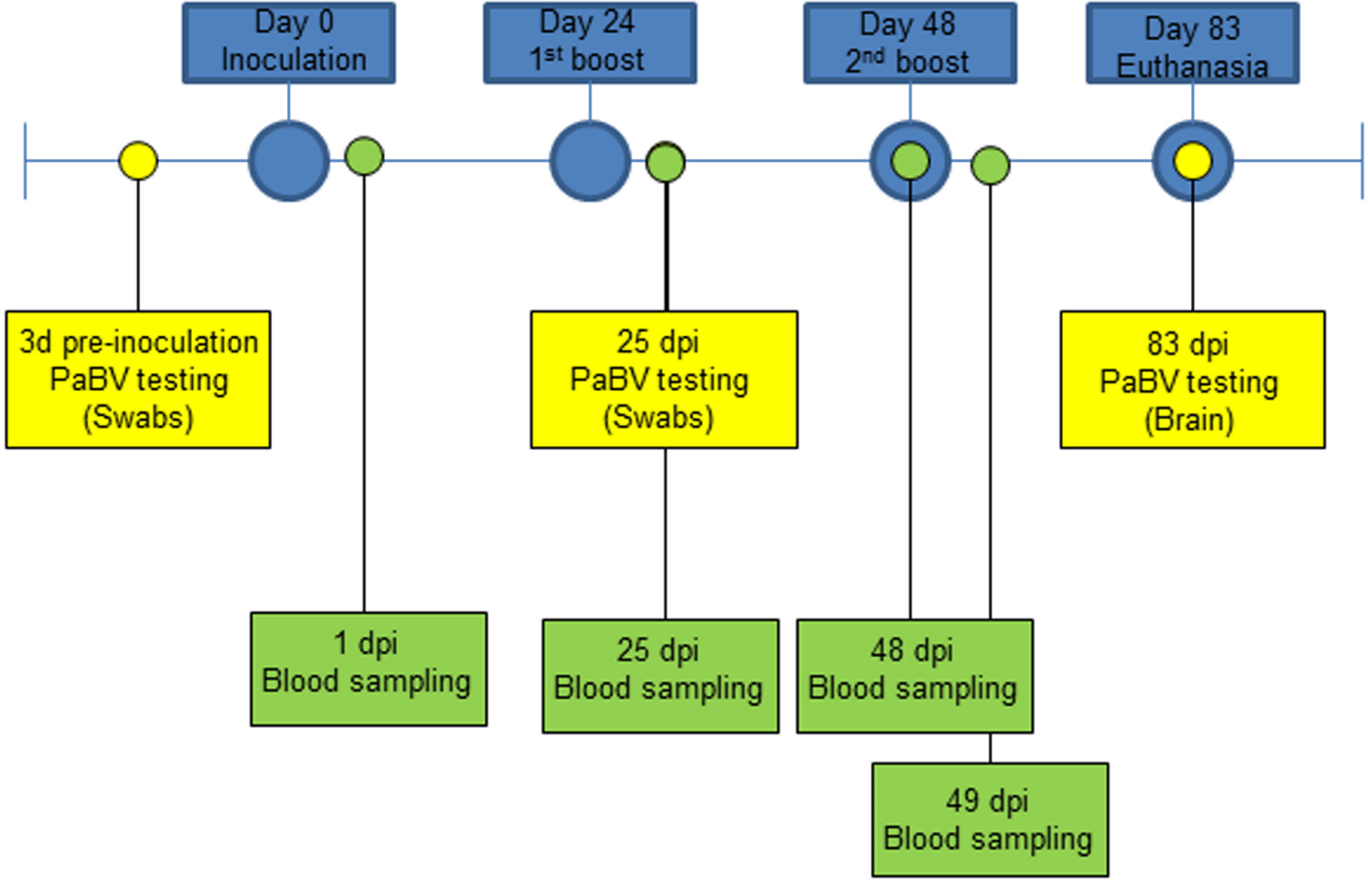
Experiment timeline for quaker parrots. They were divided into 3 groups and inoculated 3 times with 0.5 ml of three different preparations (Purified gangliosides + Freund’s adjuvant; Crude nervous tissues + Freund’s adjuvant; or only Freund’s adjuvant) with 24 days between each inoculation. RT-PCR for PaBV was performed from cloacal swabs at 3 dpi, 25 dpi and from brain at 83 dpi. Blood samples were collected at 1 dpi, 25 dpi, and 48 dpi and were used for serological and fibrinogen analysis, and at 49 dpi to evaluate fibrinogen levels following the second ganglioside inoculation.

The second experiment included 5 healthy adult quaker parrots (*Myiopsitta monachus*) selected from a colony tested for PaBV every three months by RT-PCR and Western blot, as previously described (Guo et al., 2014), and were consistently negative at all times. The quaker parrots were divided into 3 groups and a solution was injected in the pectoral muscle, as follows: group 1, one quaker parrot (QP1) was inoculated with 0.5 ml of FCA (negative control); group 2, two quaker parrots (QP2 and QP3) were inoculated with 0.5 ml of an emulsion of FCA and purified quaker brain gangliosides; and group 3 (QP4 and QP5) consisted of two quaker parrots inoculated with 0.5 ml of an emulsion of FCA and crude nervous extracts (mixture of brain, spinal cord, sciatic, and brachial nerves, and nerve of Remak). All quaker parrots were inoculated three times, with 24 days interval between each inoculation and euthanized at 83 dpi (Fig. 1). In contrast to the chickens, an additional inoculation was performed in the quaker parrots in order to stimulate the production of antibodies. All animals in this experiment were anesthetized with Isofluorane and then euthanized with carbon dioxide (CO_2_). All birds were clinically evaluated daily and any abnormal signs (particularly neurological or gastrointestinal signs) or behavior were documented. Blood samples from quaker parrots were collected at different time points for Western blot, ELISA and fibrinogen determination (Fig. 1).

### RT-PCR

The quaker parrots used in this experiment were further tested for PaBV by RT-PCR. Cloacal and choanal swabs were collected for RT-PCR 3 days before inoculation and 25 dpi. Samples from brain, spinal cord and nerves were also collected during necropsy at 83 dpi and tested by RT-PCR. Briefly, RNA was extracted from samples using a QIAamp^®^ Viral RNA Mini Kit (Qiagen, Valencia, CA, USA) and reverse-transcribed and amplified using primers targeting the PaBV matrix gene, as previously described (Guo et al., 2014). Positive and negative samples from other psittacine birds not related to this study were used as positive and negative controls. The amplification products were analyzed by a 1.5% agarose gel electrophoresis. The nervous tissues used for the preparation of the inoculum were also tested by RT-PCR for PaBV prior to inoculation into the quaker parrots.

### Fibrinogen assessment

Plasma samples from quaker parrots were submitted to the Clinical Pathology laboratory at the Veterinary Medicine Teaching Hospital at Texas A&M University for measurement of fibrinogen concentrations using the heat precipitation method as previously described (Millar, Simpson & Stalker, 1971). Samples were collected at 1 dpi, 25 dpi, 48 dpi and 49 dpi (Fig. 1).

### Pathology

A systematic necropsy was performed on all birds and representative tissues of CNS, PNS, gastrointestinal tract, skin, musculoskeletal, endocrine and genitourinary systems were collected and processed for routine histological examination.

### Serology

Quaker parrot sera were evaluated for reactivity against the crude and purified extracts used for inoculation by Western blot. This technique was performed based on previously described methods (Guo et al., 2014; Towbin et al., 1979). Briefly, crude and purified extracts were mixed with gel loading buffer and electrophoresed on 10% polyacrylamide gels. Proteins were transferred to a polyvinylidene fluoride (PVDF) membrane in transfer buffer at 100 mA for 2 h. Membranes were incubated in blocking solution at room temperature for 2 h and then cut into strips and incubated for 2 h with each individual bird serum diluted 1:1,000 in blocking solution and acting as the primary antibody. The membrane stripes were then incubated with alkaline phosphatase labeled goat anti-macaw IgY antibody diluted 1:5,000 in blocking buffer for 1 h. Membranes were rinsed and stained with 5-bromo-4-chloro-3-indolyl phosphate/p-nitroblue tetrazolium chloride (BCIP/NBT) (Sigma-Aldrich, St.Louis, MO, USA).

Measurement of anti-gangliosides antibodies was performed using enzyme-linked immunosorbent assay as previously described (Alaedini et al., 2002), with minor modifications for use with bird serum. Sera from 1 dpi, 25 dpi and 48 dpi from each quaker parrot was analyzed by ELISA and a healthy macaw serum was also used as negative control. Microtiter plates were coated with 100 µl per well of the crude or purified extracts in triplicate, and incubated at 4 °C overnight. Following incubation, wells were blocked with 5% fat-free dried milk in PBST for 1 h. Serum from each bird was diluted 1:5,000 and added to the wells and incubated for 1 h at room temperature. After three washes of PBST, the plates were incubated for 1 h with 100 µl of anti-macaw IgY antibody. TMB (3,3′, 5,5;-tetramethylbenzidine) substrate (Invitrogen, Carlsbad, CA) was added and incubated for 15 min. Sulfuric acid (H_2_SO_4_) was used to stop the reaction and optical density (OD) was measured at 450 nm. Sera with absorbance values higher than 0.1 were considered positive.

## Results

### Thin Layer Chromatography (TLC)

Using a solvent system comprised by chloroform/methanol/0.2% aqueous CaCl_2_ 50:42:11 volume, we were able to separate the aqueous phase of the inoculum demonstrating the presence of gangliosides when compared to the standard bovine ganglioside mixture. Using Ehrlich’s reagent, gangliosides reacted as grey spots on the TLC plate.

### Clinical signs

Chicken C1 presented with mild walking difficulty and weakness at 14 dpi and was euthanized. All of the other chickens remained healthy and were euthanized at the end of the experimental period at 62 dpi. None of the quaker parrots presented clinical signs that resembled PDD (i.e., undigested seeds in the feces, regurgitation, neurological signs) or a peripheral neuropathy that could resemble GBS. However, nonspecific signs such as mild depression (QP2 and QP5) and weight loss (QP4) were observed.

### PaBV-RT-PCR

All quaker parrots tested negative for PaBV on cloacal and choanal swabs by RT-PCR 3 days before inoculation and 25 dpi. Samples collected from the brain, spinal cord and nerves from all quaker parrots at time of euthanasia, as well as the inoculum used to inject the birds, were negative for PaBV by RT-PCR.

### Fibrinogen analysis

Fibrinogen levels, an important inflammatory marker in birds, were increased in QP5, 24 h after each inoculation, reaching levels up to 400 mg/dL (Table 1). QP2, QP3, QP4 had variable fibrinogen levels, with the highest levels at 49 dpi. The fibrinogen levels for control QP1 were less than 100 mg/dL throughout the study.

**Table 1:** Fibrinogen levels (mg/dL) in quaker parrots measured at 4 different timepoints.

QP#	1 dpi	25 dpi	48 dpi	49 dpi
1	<100	<100	<100	100
2	<100	100	<100	200
3	<100	<100	<100	400
4	100	<100	100	400
5	200	300	300	400

### Serology

**Figure 2:**
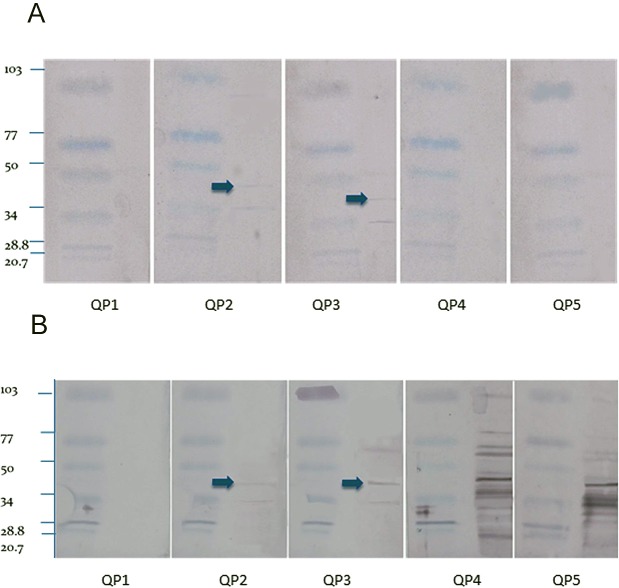
Western blot. Purified (A) and crude (B) nervous extracts exposed to sera from 5 different quaker parrots. Bird 1 was inoculated only with Freund’s adjuvant and served as control. Quaker parrots 2 and 3 were inoculated with purified gangliosides and quaker parrots 4 and 5 were inoculated with crude nervous tissue extracts. Quaker parrots 2 and 3 presented bands with approximately 45 kD (arrows), while the crude extract blots presented several unspecific bands when exposed to QP4 and QP5 sera.

The Western blot using the crude extract showed fewer bands from the sera from the quaker parrots inoculated with the purified ganglioside extract (QP2 and QP3) and several nonspecific bands for the birds inoculated with the crude extract (QP4 and QP5) (Fig. 2). The Western blot from control QP1 did not present any bands. The blots loaded with the purified extract only reacted with the sera of the birds inoculated with the purified extract, showing fewer faint bands when compared to the crude extract blots. A 45 kDa band was observed in the blots from QP2 and QP3, which were treated with purified gangliosides. This is the molecular weight of ganglioside binding proteins (Riggott & Matthew, 1996).

**Figure 3:**
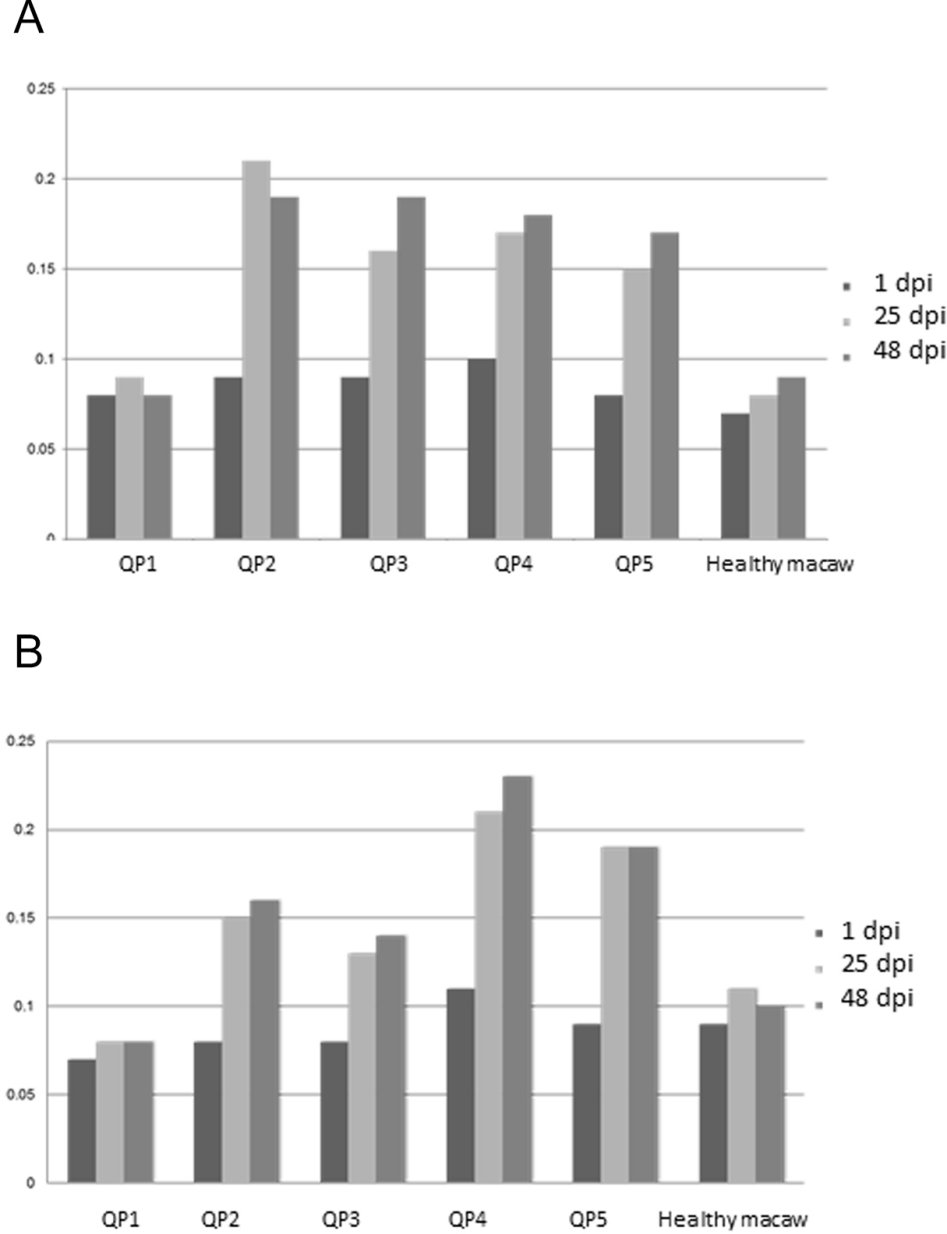
Enzyme-linked immunosorbent assay. Variability of auto-ganglioside antibodies of each quaker parrot serum measured by ELISA for three different timepoints. (A) Plate coated with purified gangliosides. (B) Plate coated with crude extracts. The vertical axis demonstrates the absorbance readings values at 450 nm. A healthy macaw serum was used as a negative control.

All inoculated quaker parrots (QP2–QP5) presented a marked increase in the absorbance values on ELISA (Fig. 3) using the purified and crude ganglioside extracts when comparing the first timepoint to the second and third timepoints. Due to the lack of a positive control originated from a quaker parrot with proved anti-ganglioside antibodies, the ELISA evaluation was performed based on the exposure of QP1 serum to the crude and purified extracts and also the serum from a healthy macaw exposed to these extracts. For the plates coated with the purified extract, QP2 had absorbance values, almost two times higher than sera from QP1, when comparing the second and third timepoints. The plates coated with crude extract, QP4 had absorbance values from the second and third timepoints almost three times higher when compared to QP1 (control). The sera from QP1 and the healthy macaw sera had constant low values at all 3 timepoints for plates coated with purified and crude extracts.

### Pathology

**Figure 4:**
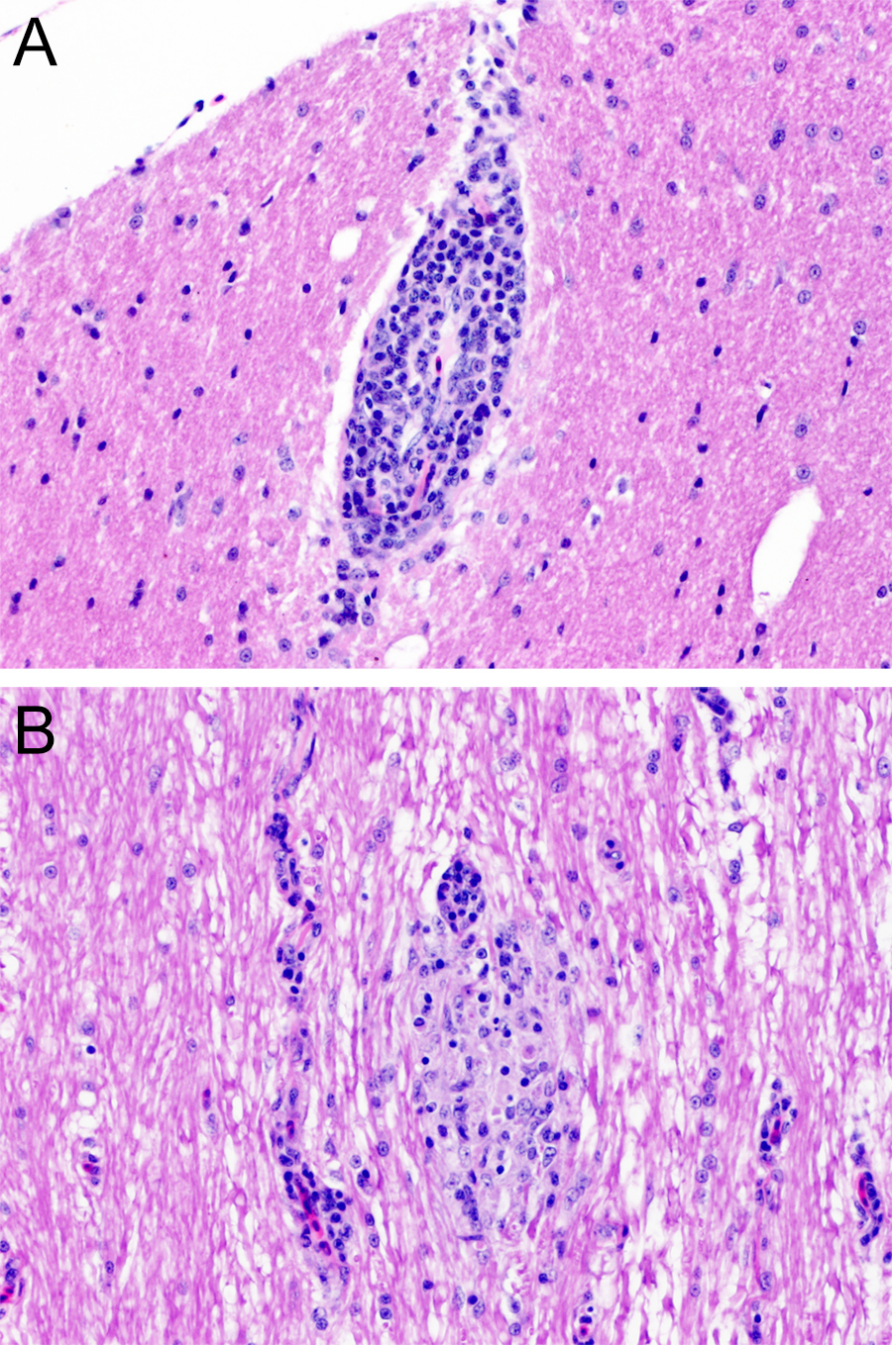
Histopathology. (A) Chronic, mild, multifocal lymphoplasmacytic encephalitis in quaker parrot 2. (B) Quaker parrot 4 had a chronic, mild, multifocal lymphoplasmacytic and hystiocytic myelitis.

Chicken C1 (euthanized at 14 dpi) had an intense inflammatory reaction in the site of the inoculation in the pectoral muscle. No gross lesions were observed in any of the quaker parrots. Histologically, C1, presented with a mild perivascular and perineural lymphocytic infiltrate in the proventriculus, while C5, and C6 presented with minimal to mild, focal, lymphocytic aggregates in the cerebrum. QP2 had a mild, multifocal, lymphoplasmacytic encephalitis in the optic tectum (Fig. 4A). QP4 had a mild, multifocal lymphoplasmacytic and histiocytic myelitis in the thoracic and lumbar segments (Fig. 4B).

## Discussion

Despite numerous studies published on PDD and its association with PaBV, the pathogenesis of this disease remains unclear. Based on studies in rats inoculated with Borna disease virus (BDV), a T cell-induced immunopathological mechanism has been favored (Hallensleben et al., 1998) for lesion development in the central and autonomic nervous system of birds inoculated with PaBV; however, few investigators have speculated an autoimmune component might play an important role in PDD pathogenesis (Graham, 1991; Rossi, Crosta & Pesaro, 2008). Previous studies developed (Rossi, Crosta & Pesaro, 2008; Pesaro, 2008), suggested that immunization of birds with gangliosides could trigger the development of PDD-like lesions.

In our first study, only one chicken (C1), presented with mild difficulty walking after 14 dpi. The clinical signs were likely related to the inflammatory reaction in the site of inoculation, characterized by a locally extensive granulomatous myositis and steatitis extending to the air sacs. Besides the lesion in the site of inoculation and air sacs, the only histologic lesion seen in this chicken was a lymphocytic infiltrate observed surrounding (but not within) one ganglion and around a vessel of the proventriculus. This lymphocytic infiltrate was interpreted as a lymphoid aggregate, occasionally observed in the gastrointestinal tract of avian species (Casteleyn et al., 2010). No histologic lesions were detected in the CNS or PNS of this chicken. Few lymphocytic aggregates were observed in the brain of two chickens These random infiltrates of mononuclear cells are also occasionally seen in the brain of otherwise healthy chickens (Abdul-Aziz, Fletcher & Barnes, 2016).

The clinical signs in quaker parrots were only observed right after the inoculation of gangliosides (mild depression) and were attributed to an expected reaction to the foreign substances. Histologic lesions were observed in only two quaker parrots and were limited to the CNS. They included a mild, multifocal, lymphoplasmacytic encephalitis (QP2) and a mild, multifocal, lymphoplasmacytic myelitis (QP4). These two quaker parrots also had the highest levels of anti-ganglioside antibodies by ELISA and showed bands compatible with the molecular weight of ganglioside binding proteins on Western blots. Quaker parrot 3 also presented a similar band, but did not have any gross or histologic lesions. All quaker parrots tested negative for PaBV by RT-PCR suggesting that these lesions were provoked by the inoculations rather than a viral agent. We cannot discard the possibility of other nervous components have caused the myelitis in QP4 since this bird was inoculated with non-purified nervous crude extracts. This finding corroborates with the hypothesis that the inoculations might have triggered an autoimmune response, rather than an immunomediated activity, as seen in PDD cases (Piepenbring et al., 2012). Most experimental studies involving PaBV inoculation in psittacine birds, were able to induce the first clinical signs of PDD, including depression, regurgitation and undigested seeds in the feces, between 21 and 66 dpi (Gancz et al., 2009; Gray et al., 2010; Piepenbring et al., 2012; Piepenbring et al., 2016). Additionally, dilation of the proventriculus associated with myenteric ganglioneuritis is a consistent lesion observed in all experimental birds that presented encephalomyelitis and also in most of the natural cases of PDD (Lierz et al., 2009; Kistler et al., 2010; Encinas-Nagel et al., 2014), which supports that if the experimental inoculation of gangliosides is directly related to the development of PDD signs, these classical neurological and gastrointestinal signs and lesions should have been observed during the 83 days of our experiment.

The hallmark of GBS is a peripheral neuropathy with limb weakness, a feature not observed in our experiment (and not a feature of PDD). Although birds unable to walk or perch have been observed in cases of PDD (Payne et al., 2011; Rubbenstroth et al., 2014), inflammatory infiltrates affecting the peripheral nerves responsible for the innervation of the limbs, are not a consistent lesion of PDD (Berhane et al., 2001; Payne et al., 2011). Because encephalomyelitis is one of the common lesions of PDD, a central disorder is more likely to be the cause of impaired deambulation. GBS lesions from nerve biopsies consist of endoneurial and epineurial infiltrates of CD3+ T cells and CD68+ macrophages. Similar lesions are observed in experimental allergic neuritis induced in Lewis’ rats, a well-established model of human autoimmune neuropathies (Shin et al., 2013). Autoreactive T lymphocytes are known to be key players in autoimmune diseases and they can be either regulatory and effector cells (Dornmair et al., 2003). These cells can be activated by several mechanisms including molecular mimicry, and viral and bacterial superantigens (Wucherpfennig, 2001). The role of autoreactive T lymphocytes has not been studied in PDD so far, and the importance of T-cell immunopathological mechanisms has only been extrapolated from studies in rats experimentally infected with BDV (Hausmann, Schamel & Staeheli, 2001; Planz, Bilzer & Stitz, 1995; Richt et al., 1994). These studies indicated that the immunopathology of BDV is mediated by CD8^+^ T cells which require help from CD4^+^ T cell subsets. These CD8^+^ T cells have shown active cytolytic activity against target cells infected with BDV or expressing BDV antigens (Schmidt et al., 1996). Macrophages have an essential role in the development of nerve lesions in GBS cases, being associated with axonal demyelination (Asbury, Arnason & Adams, 1969; Griffin et al., 1996a; Griffin et al., 1996b; Prineas, 1972; Staeheli, Rinder & Kaspers, 2010). In birds, the lesions caused by PaBV consist of a lymphoplasmacytic infiltrate in either the CNS or PNS (Kistler et al., 2008; Staeheli, Rinder & Kaspers, 2010) and demyelination is not a feature of PDD.

Published studies involving experimental inoculation of PaBV in psittacine birds and following the premises of Koch’s postulates (Gancz et al., 2009; Gray et al., 2010; Payne et al., 2011; Piepenbring et al., 2012; Rubbenstroth et al., 2014) have been able to induce clinical disease and cause CNS and PNS inflammation in most inoculated birds, supporting the viral etiology of PDD. This data corroborates with the hypothesis that PaBV, like other neurotropic viruses, such as Lyssavirus, directly invades the nervous tissues and triggers inflammation and consequently provokes the damage in the nervous system. Nonetheless, in some diseases such as human idiopathic achalasia, antineuronal antibodies are generated following nervous tissue damage and do not directly influence the development of the disease (Moses et al., 2003; Storch et al., 1995). A similar association has been proposed for the recent cases of human fatal encephalitis associated with variegated squirrel 1 bornavirus (Hoffmann et al., 2015; Tappe, Schmidt-Chanasit & Beer, 2015).

In our study, quaker parrots inoculated with gangliosides and examined 83 dpi had no signs of myenteric ganglioneuritis, which differs from experimental studies with PaBV and corroborates with the hypothesis that anti-ganglioside antibodies are not involved in the pathogenesis of PDD. Our results suggest that an autoimmune response to brain gangliosides does not cause the development of PDD-like lesions, and supports that an immune-mediated response might be associated with the pathogenesis of this disease. Additionally, psittacine birds experimentally inoculated with gangliosides and nervous tissues extracts are not an appropriate animal model for GBS. Further studies are needed in order to analyze the participation of anti-gangliosides antibodies in natural cases of PDD and in birds experimentally inoculated with PaBV.

##  Supplemental Information

10.7717/peerj.3144/supp-1 Data S1 ELISA raw data

## Additional Information and Declarations

### Data Availability

The following information was supplied regarding data availability: The raw data has been supplied as Data S1.
